# Serum vitamins and *Mycoplasma pneumoniae* pneumonia in children: a case-control study

**DOI:** 10.3389/fimmu.2026.1676950

**Published:** 2026-02-25

**Authors:** Tiewei Li, Nan Chen, Xucheng Wang, Xiaojuan Li, Panpan Fang, Junmei Yang, Zhipeng Jin

**Affiliations:** 1 Department of Clinical Laboratory, Zhengzhou Key Laboratory of Children’s Infection and Immunity, Children’s Hospital Affiliated to Zhengzhou University, Zhengzhou, China; 2 Henan Children’s Hospital, Zhengzhou Children’s Hospital, Zhengzhou, China; 3 Pediatric Intensive Care Unit, Children’s Hospital Affiliated to Zhengzhou University, Zhengzhou, China

**Keywords:** children, *Mycoplasma pneumoniae* pneumonia, vitamin A, vitamin B1, vitamin C, vitamin D, vitamin B7

## Abstract

**Background/objective:**

Immunodeficiency is a common precipitating factor for *Mycoplasma pneumoniae* pneumonia (MPP). Vitamins are essential for enhancing immune function and mitigating systemic inflammation. However, the relationship between various vitamins, particularly B vitamins, and MPP in children remains underexplored. This study aims to assess the nutritional status of multiple vitamins in children with MPP and their relationship to the condition.

**Methods:**

A retrospective observational study was conducted at Children’s Hospital Affiliated to Zhengzhou University. A total of 135 children diagnosed with MPP with voluntarily requested vitamin profiling were enrolled between October and December 2023. A control group of 199 children, who underwent health check-ups and vitamins assessments, were also included during the same period. Clinical and laboratory data were retrieved from the hospital’s electronic medical record system.

**Results:**

Children with MPP exhibited significantly lower levels of VA, VD, VB1, VB7, and VC in their peripheral blood compared to the healthy control group. Nutritional analysis revealed higher deficiency rates of these vitamins in the MPP group. Correlation analysis indicated significant negative relationships between VA, VD, VB1, VB7, and VC levels and the percentage of neutrophils. Additionally, VA, VD, VB7, and VC levels were negatively correlated with the percentage of monocytes. Multivariate regression analysis, adjusted for age and neutrophil percentage, showed that VA (OR = 0.986, 95% CI: 0.981-0.992, P < 0.001), VD (OR = 0.807, 95% CI: 0.746-0.874, P < 0.001), VB1 (OR = 0.592, 95% CI: 0.406-0.864, P = 0.007), VB7 (OR = 0.980, 95% CI: 0.972-0.989, P < 0.001), and VC (OR = 0.899, 95% CI: 0.822-0.984, P = 0.021) were independently associated with MPP. Further analysis demonstrated that children with deficiencies in VA, VD, VB1, VB7, and VC had significantly higher odds of having MPP.

**Conclusion:**

Children with MPP exhibit significantly lower levels of VA, VD, VB1, VB7, and VC. The incidence of multiple vitamin deficiencies is notably higher in this group compared to healthy children, and a negative correlation exists between vitamin levels and neutrophil percentage. Multivariate regression analysis confirms that VA, VD, VB1, VB7, and VC were identified to be independently associated with MPP.

## 1 Introduction

*Mycoplasma pneumoniae* (*MP*) is a predominant respiratory pathogen with a high prevalence among pediatric and adolescent populations (1). Following the 2023 COVID-19 pandemic and the subsequent relaxation of non-pharmaceutical interventions (NPIs), a marked surge in acute respiratory infections among Chinese children has been observed, with *MP* emerging as the most frequently detected pathogen. In several regions, positivity rates have exceeded 50% (2). *Mycoplasma pneumoniae* pneumonia (MPP), induced by *MP*, manifests as pulmonary inflammation involving the bronchi, bronchioles, alveoli, and interstitial tissues, constituting a significant etiology of pediatric pneumonia (3). Clinical presentations typically include fever and cough, often accompanied by headache, rhinorrhea, pharyngalgia, otalgia, and wheezing (4). Recent epidemiological trends indicate an increasing incidence among younger age groups, notably preschool-aged children (5, 6). Simultaneously, the prevalence of refractory MPP (RMPP) and macrolide-resistant strains has escalated (1), presenting substantial challenges to pediatric healthcare and imposing considerable socioeconomic burdens. Despite the increasing incidence, current preventive and therapeutic approaches remain inadequate.

Vitamins, categorized into water-soluble and fat-soluble types, are fundamental micronutrients essential for maintaining physiological health, with each vitamin subtype playing distinct roles in energy metabolism, antioxidant mechanisms, immune modulation, and hemostasis (7–9). Deficiencies in vitamins are strongly correlated with the onset and progression of various infectious diseases. In recent years, the immunomodulatory properties of vitamins have garnered substantial attention. Vitamin D (VD) deficiency, in particular, has been closely linked to compromised immune function in pediatric populations (10). VD exerts regulatory effects on immune cell activity, including the activation and proliferation of T and B lymphocytes (11). Insufficient VD levels are associated with diminished immune responsiveness to infections, predisposing affected children to increased infection risk and more severe disease courses (12) (13). Similarly, Vitamin A (VA) is integral to immune competence, particularly in maintaining mucosal immunity (14, 15). VA deficiency may impair the differentiation and function of immune cells, thereby reducing pathogen recognition and clearance capacity (16). Furthermore, B vitamin deficiencies have been implicated in altered immune responses in children. Notably, vitamin B6 has demonstrated anti-inflammatory properties by attenuating lipopolysaccharide (LPS)-induced inflammation (17). Moreover, B vitamins collectively influence gut microbiota composition, thereby modulating immune function (18).

Despite the recognized importance of vitamins in immune regulation, the relationship between vitamin levels and MPP remains insufficiently investigated, especially concerning B vitamins. This study aims to systematically evaluate the levels and nutritional status of multiple vitamins in children diagnosed with MPP utilizing ultra-high-performance liquid chromatography-tandem mass spectrometry (UHPLC-MS/MS). A comparative analysis with healthy children undergoing routine physical examinations will be conducted to elucidate the potential association between vitamin status and MPP.

## 2 Materials and methods

### 2.1 Study population

A retrospective case-control study was conducted at Children’s Hospital Affiliated to Zhengzhou University. The case group consisted of 135 hospitalized children diagnosed with MPP who were systematically recruited between October and December 2023. Vitamin levels for these patients were assessed based on voluntary requests as part of their clinical management. The diagnosis was confirmed by a positive polymerase chain reaction (PCR) test for *MP* nucleic acid from throat swabs, in accordance with the “Diagnosis and Treatment Guidelines for *Mycoplasma Pneumonia* in Children (2023 Edition)”. The inclusion criteria for the MPP cases were as follows: (1) age >1 year; and (2) availability of complete clinical data, including chest imaging and laboratory test results, within the electronic medical record system. Exclusion criteria were applied to ensure a homogenous study population and minimize confounding: (1) history of chronic conditions such as tuberculosis, chronic cardiopulmonary diseases, or disorders impairing airway mucociliary clearance (e.g., cystic fibrosis, primary ciliary dyskinesia); (2) congenital bronchial or pulmonary malformations; (3) immunodeficiency or other immune-related disorders; (4) confirmed bacterial co-infections upon hospital admission; and (5) prior receipt of vitamin supplementation.

To ensure comparability, the control group was selected from the same hospital setting. It comprised 199 healthy children who were consecutively recruited from the pediatric health clinic during the same period (October to December 2023), thus matching the cases on the key parameter of season and location. Controls were defined as children attending the clinic for routine health check-ups. The inclusion criteria for controls were: (1) age >1 year (to match the case group); and (2) availability of complete clinical and laboratory test data relevant to the study. The exclusion criteria for the control group were strictly applied to exclude any children with active illness or underlying conditions: (1) presence of any symptoms suggestive of acute respiratory infection at the time of recruitment; (2) white blood cell or neutrophil counts outside the normal age-specific reference range; (3) any of the conditions listed in the exclusion criteria for the MPP cases (e.g., chronic cardiopulmonary diseases, immunodeficiencies).

The study protocol, approved by the Ethics Review Committee of Henan Children’s Hospital (Approval No. 2022-K-L045), was in accordance with the Declaration of Helsinki. All procedures, including the vitamin level assessments which were performed at the request of the patients or their guardians as part of clinical care, were part of routine practice. Data collection was retrospective, with strict anonymization protocols ensuring confidentiality. The requirement for informed consent was waived by the Ethics Review Board due to the retrospective nature of the study (Approval No. 2022-K-L045).

### 2.2 Diagnosis of MPP

The diagnostic criteria for MPP in children followed the “Diagnosis and Treatment Guidelines for *Mycoplasma Pneumonia* in Children (2023 Edition)” (3). Diagnosis was established based on clinical and imaging features in conjunction with one or more of the following: (1) a serum *MP* antibody titer ≥ 1:160; (2) a fourfold or greater increase in serum *MP* antibody titer from paired samples during illness; or (3) positive *MP* DNA or RNA tests. In this study, MPP was confirmed by positive *MP* nucleic acid testing using the *MP* nucleic acid detection kit (Jiangsu Mole Biotechnology Co., Ltd) from throat swabs. Diagnoses were independently verified by two clinicians based on the clinical presentation.

### 2.3 Data collection

Clinical and laboratory data from both subjects with MPP and healthy controls were extracted from the hospital’s electronic medical records, including demographic data (sex, age) and hematological indices. Initial complete blood count results and vitamin levels (vitamin A, D, E, B1, B2, B3, B5, B7, and C, and 5-methyltetrahydrofolate) obtained within 24 hours of hospitalization were analyzed for both groups. Vitamin testing is performed based on clinical needs and parental requests as determined by healthcare providers. The detailed methodologies for vitamin detection are provided in the **Supplementary Materials**. The method validation demonstrated excellent analytical performance: (1) intra-day precision ≤10.60% (n=20), (2) inter-day precision ≤8.10% (3 consecutive days), and accuracy within 85.05-114.79% recovery across all tested vitamins, meeting FDA bioanalytical method validation criteria. Hematological profiles were determined using a UPPER fully automated hematology analyzer (Shanghai Aopu Bio-Pharmaceutical Co., Ltd.), measuring neutrophil, lymphocyte, and monocyte percentages. In pediatric hematology, age-stratified reference ranges for leukocyte differential percentages are defined as follows: Neutrophil percentages demonstrate progressively increasing values from 13–54% (1 to <2 years) to 23–64% (2 to <6 years), 32–71% (6 to <13 years), and 33–74% (13 to <18 years). Conversely, lymphocyte percentages exhibit declining ranges: 35–76% (1 to <2 years), 26–67% (2 to <6 years), 22–57% (6 to <13 years), and 20–54% (13 to <18 years). Monocyte percentages remain relatively stable at 2–14% in children aged 1 to <2 years, narrowing to 2–11% through ages 2 to <18 years.

### 2.4 Definitions of vitamin deficiency

According to the “Expert Consensus on Clinical Application of Vitamin A and Vitamin D for Children in China (2024),” (3),VA deficiency is defined as levels below 300 ng/ml, and VD deficiency as levels below 20 ng/ml.

Currently, there are no uniform international or domestic guidelines defining deficiency thresholds for vitamin E (VE), vitamin C (VC), and B vitamins. Therefore, the thresholds for insufficiency used in this study were established based on reference ranges provided by leading clinical laboratories, including Mayo Clinic, Labcorp, and Quest Diagnostics.

- VE deficiency: < 3.8 μg/mL (Mayo Clinic).
- VB1 deficiency: < 2.12 ng/mL (Quest Diagnostics).
- VB2 deficiency: < 1 ng/mL (Mayo Clinic).
- VB3 deficiency: < 5.2 ng/mL (Labcorp).
- VB5 deficiency (Mayo Clinic):
  - Ages 0–10 years: < 3.45 ng/mL.
  - Ages > 10 years: < 37 ng/mL.
- VB7 deficiency (Mayo Clinic):
  - Ages < 12 years: < 0.1 ng/mL.
  - Ages ≥ 12 years: < 0.22 ng/mL.
- 5-methyltetrahydrofolate (5-MTHF) deficiency: < 4 ng/mL (Mayo Clinic).
- VC insufficiency: < 4 μg/mL (Mayo Clinic).

### 2.5 Statistical analysis

All statistical analyses were performed using SPSS version 24.0 (IBM Corp., Armonk, NY). Continuous variables were first assessed for normality via the Shapiro-Wilk test. Normally distributed data were expressed as mean ± standard deviation, and comparisons between two groups were performed using t-tests. Non-normally distributed data are presented as median (interquartile range [IQR]) and analyzed using the Mann-Whitney U test. Categorical variables (e.g., sex, vitamin deficiency status) are expressed as frequency (percentage) and compared via a Chi-square test. To account for multiple comparisons in the analysis of multiple vitamins, the false discovery rate (FDR) correction was applied using the Benjamini-Hochberg procedure. Correlations between vitamin levels and clinical/laboratory parameters were evaluated using Spearman’s rank correlation coefficient. Univariate logistic regression identified independent factors for MPP. Variables showing significant associations (P < 0.05) in univariate analysis were subsequently included in a multivariable logistic regression model to determine independent predictors. A two-sided P-value < 0.05 was considered statistically significant for all analyses.

## 3 Results

### 3.1 Study population characteristics

Between October and December 2023, 135 children with positive *MP* nucleic acid results confirmed by PCR were enrolled in this study, alongside 199 healthy children serving as the control group. As presented in **Table 1**, compared with healthy children, children with MPP had higher body temperature, respiratory rate, and heart rate. No significant differences in age, sex, height and weight were observed between the two groups. Additionally, there was no significant difference in residential classification (urban vs. rural) between the MPP patients and healthy controls. A further analysis of complete blood count data revealed that the percentages of neutrophils and monocytes in the peripheral blood of the MPP group were significantly elevated compared to those in the healthy control group, while the percentage of lymphocytes was notably lower in the MPP group.

**Table 1:** Basic characteristics of study participants in control and MPP groups.

Variables	Control (n = 199)	MPP (n = 135)	P
Age (years)	7.0 (4.9, 9.0)	7.0 (4.9, 9.0)	0.305
Male, n (%)	107 (53.8%)	72 (53.3%)	0.938
Temperature (°C)	36.3 ± 0.8	36.9 ± 0.7	< 0.001
Respiratory rate (rate/min)	20.6 ± 1.8	25.4 ± 4.4	< 0.001
Heart rate (rate/min)	88.4 ± 10.0	101.5 ± 13.2	< 0.001
Height (cm)	127.5 (111.0, 139.5)	130.0 (110.0, 140.0)	0.952
Weight (kg)	24.1 (17.5, 35.7)	24.8 (18.5, 32.2)	0.898
Neutrophil (%)	46.0 (40.6, 56.0)	64.7 (54.6, 74.3)	< 0.001
Monocyte (%)	2.9 (2.5, 3.3)	4.3 (3.0, 5.7)	< 0.001
Lymphocyte (%)	46.5 (37.7, 53.4)	29.2 (19.6, 37.6)	< 0.001
Residential classification			0.556
Urban	35 (17.6%)	27 (20.0%)	
Rural	164 (82.4%)	108 (80.0%)	

MPP, *Mycoplasma pneumoniae* pneumonia.

### 3.2 Vitamin levels and nutritional status in two groups of children

Analysis of peripheral blood vitamin levels in both groups revealed that the concentrations of VA, VD, VB1, VB7, and VC in the MPP group were significantly lower than those in the healthy control group (**Table 2**). Additionally, the deficiency rates for each vitamin were markedly higher in the MPP group compared to the control group. Specifically, the deficiency rates were as follows: VA (71.9% vs. 14.1%, P < 0.001), VD (73.3% vs. 25.6%, P < 0.001), VB1 (57.0% vs. 21.6%, P < 0.001), VB7 (92.6% vs. 62.8%, P < 0.001), and VC (35.6% vs. 8.5%, P < 0.001) (**Table 3**).

**Table 2:** Comparison of multiple vitamin levels between two groups of children.

Variables	Control (n = 199)	MPP (n = 135)	P	FDR P value
VA (ng/ml)	376.3 (330.5, 458.4)	245.5 (193.3, 322.0)	< 0.001	< 0.001
VD (ng/ml)	24.9 (19.8, 29.9)	14.7 (11.4, 20.5)	< 0.001	< 0.001
VE (μg/ml)	6.4 (5.4, 7.4)	6.3 (5.5, 7.6)	0.832	0.924
VB1 (ng/ml)	2.8 (2.2, 3.7)	2.0 (1.6, 2.7)	< 0.001	< 0.001
VB2 (ng/ml)	9.4 (6.6, 13.4)	9.5 (7.9, 14.1)	0.051	0.085
VB3 (ng/ml)	31.0 (20.9, 43.8)	31.1 (20.6, 40.6)	0.958	0.958
VB5 (ng/ml)	60.6 (47.2, 78.1)	64.2 (51.8, 85.5)	0.054	0.085
VB7 (pg/ml)	84.0 (45.0, 122.0)	8.0 (1.0, 45.0)	< 0.001	< 0.001
5-MTHF (ng/ml)	10.6 (6.9, 18.4)	11.8 (7.9, 19.2)	0.137	0.171
VC (μg/ml)	9.7 (6.2, 12.9)	5.7 (2.9, 8.8)	< 0.001	< 0.001

P values were adjusted for multiple comparisons using the Benjamini-Hochberg FDR procedure.

MPP, *Mycoplasma pneumoniae* pneumonia, VA, vitamin A, VD, vitamin D, VE, vitamin E, VB1, vitamin B1, VB2, vitamin B2, VB3, vitamin B3, VB5, vitamin B5, VB7, vitamin B7, 5-MTHF, 5-methyltetrahydrofolate, VC, vitamin C.

**Table 3:** The nutritional status of multiple vitamins in the control and MPP groups.

Variables	Control (n = 199)	MPP (n = 135)	P	FDR P value
VA status			< 0.001	< 0.001
VA sufficiency, n (%)	28 (85.9%)	38 (28.1%)		
VA deficiency, n (%)	171 (14.1%)	97 (71.9%)		
VD status			< 0.001	< 0.001
VD sufficiency, n (%)	148 (74.4%)	36 (26.7%)		
VD deficiency, n (%)	51 (25.6%)	99 (73.3%)		
VE status			0.259	0.324
VE sufficiency, n (%)	192 (96.5%)	133 (98.5%)		
VE deficiency, n (%)	7 (3.5%)	2 (1.5%)		
VB1 status			< 0.001	< 0.001
VB1 sufficiency, n (%)	156 (78.4%)	58 (43.0%)		
VB1 deficiency, n (%)	43 (21.6%)	77 (57.0%)		
VB2 status			1.000	1.000
VB2 sufficiency, n (%)	199 (100.0%)	135 (100.0%)		
VB2 deficiency, n (%)	0 (0)	0 (0)		
VB3 status			< 0.001	< 0.001
VB3 sufficiency, n (%)	199 (100.0%)	135 (100.0%)		
VB3 deficiency, n (%)	0 (0)	0 (0)		
VB5 status			0.085	0.121
VB5 sufficiency, n (%)	199 (100.0%)	133 (98.5%)		
VB5 deficiency, n (%)	0 (0)	2 (1.5%)		
VB7 status			< 0.001	< 0.001
VB7 sufficiency, n (%)	74 (37.2%)	10 (7.4%)		
VB7 deficiency, n (%)	125 (62.8%)	125 (92.6%)		
5-MTHF			0.775	0.861
5-MTHF sufficiency, n (%)	181 (91.0%)	124 (91.9%)		
5-MTHF deficiency, n (%)	18 (9.0%)	11 (8.1%)		
VC status			< 0.001	< 0.001
VC sufficiency, n (%)	182 (91.5%)	87 (64.4%)		
VC deficiency, n (%)	17 (8.5%)	48 (35.6%)		

P values were adjusted for multiple comparisons using the Benjamini-Hochberg FDR procedure.

MPP, *Mycoplasma pneumoniae* pneumonia, VA, vitamin A, VD, vitamin D, VE, vitamin E, VB1, vitamin B1, VB2, vitamin B2, VB3, vitamin B3, VB5, vitamin B5, VB7, vitamin B7, 5-MTHF, 5-methyltetrahydrofolate, VC, vitamin C.

### 3.3 Correlation analysis between vitamins and clinical indicators

Correlation analysis demonstrated significant negative associations between VD and age (r = -0.492, p < 0.001) and between VB1 and age (r = -0.245, p < 0.001). Furthermore, VA, VD, VB1, VB7, and VC exhibited significant negative correlations with neutrophil percentage while showing significant positive correlations with lymphocyte percentage. Additionally, VA, VD, VB7, and VC were significantly negatively correlated with monocyte percentage (**Table 4**).

**Table 4:** Correlation between vitamin levels, age, and inflammatory markers.

Variables	VA		VD		VB1		VB7		VC	
	r	P	r	P	r	P	r	P	r	P
Age (years)	0.063	0.254	-0.492	< 0.001	-0.245	< 0.001	-0.075	0.172	-0.048	0.377
Neutrophil (%)	-0.411	< 0.001	-0.509	< 0.001	-0.317	< 0.001	-0.429	< 0.001	-0.409	< 0.001
Monocyte (%)	-0.307	< 0.001	-0.168	0.002	-0.079	0.149	-0.209	< 0.001	-0.135	0.013
Lymphocyte (%)	0.429	< 0.001	0.488	< 0.001	0.302	< 0.001	0.426	< 0.001	0.431	< 0.001

VA, vitamin A, VD, vitamin D, VB1, vitamin B1, VB7, vitamin B7, VC, vitamin C.

### 3.4 Relationship between vitamin levels, nutritional status, and MPP

Univariate regression analysis identified age, body temperature, respiratory rate, heart rate, neutrophil percentage, VA, VD, VB1, VB7, and VC as factors associated with MPP. After adjusting for age, body temperature, respiratory rate, heart rate, and neutrophil percentage, multivariate regression analysis revealed that VA (OR = 0.986, 95% CI: 0.981-0.992, P < 0.001), VD (OR = 0.807, 95% CI: 0.746-0.874, P < 0.001), VB1 (OR = 0.592, 95% CI: 0.406-0.864, P = 0.007), VB7 (OR = 0.980, 95% CI: 0.972-0.989, P < 0.001), and VC (OR = 0.899, 95% CI: 0.822-0.984, P = 0.021) were independently associated with MPP (**Table 5**). Furthermore, multivariate regression analysis revealed that children with deficient levels of VA (OR = 8.515, 95% CI: 3.586-20.219, P < 0.001), VD (OR = 12.038, 95% CI: 4.414-32.826, P < 0.001), VB1 (OR = 5.082, 95% CI: 2.211-11.677, P < 0.001), VB7 (OR = 7.313, 95% CI: 2.501-21.382, P < 0.001), and VC (OR = 3.056, 95% CI: 1.089-8.578, P = 0.034) had significantly higher odds of having MPP compared to children with sufficient vitamin levels (**Table 6**). We further investigated their combined effect. Notably, the combined VA+VD level (per ng/ml) showed a markedly strong association with MPP (OR = 380.724, 95% CI: 57.018-2542.182, P < 0.001). Children with concurrent deficiency of both vitamins A and D demonstrated an exceptionally high odds ratio for MPP (OR = 101.494, 95% CI: 21.111-487.946, P < 0.001) (**Supplementary Table 1**).

**Table 5:** Association between vitamin levels and MPP.

Variables	Univariate		Multivariate*			
	OR (95% CI)	P	OR (95% CI)	P	Nagelkerke R²	Hosmer-lemeshow test (P)
VA (ng/ml)	0.983 (0.979-0.987)	<0.001	0.986 (0.981-0.992)	<0.001	0.791	0.249
VD (ng/ml)	0.884 (0.855-0.914)	<0.001	0.807 (0.746-0.874)	<0.001	0.807	0.329
VB1 (ng/ml)	0.588 (0.472-0.732)	<0.001	0.592 (0.406-0.864)	0.007	0.749	0.245
VB7 (pg/ml)	0.975 (0.969-0.982)	<0.001	0.980 (0.972-0.989)	<0.001	0.781	< 0.001
VC (μg/ml)	0.827 (0.781-0.877)	<0.001	0.899 (0.822-0.984)	0.021	0.745	0.082

*Adjusted for age, temperature, respiratory rate, heart rate and neutrophil percentage.

MPP, *Mycoplasma pneumoniae* pneumonia, VA, vitamin A, VD, vitamin D, VB1, vitamin B1, VB7, vitamin B7, VC, vitamin C.

**Table 6:** Association of vitamin deficiency with MPP.

Variables	Univariate		Multivariate*			
	OR (95% CI)	P	OR (95% CI)	P	Nagelkerke R²	Hosmer-lemeshow test (P)
VA status						
VA sufficiency	1		1			
VA deficiency	15.589 (9.012-26.967)	<0.001	8.515 (3.586-20.219)	<0.001	0.779	0.003
VD status						
VD sufficiency	1		1			
VD deficiency	7.980 (4.856-13.115)	<0.001	12.038 (4.414-32.826)	<0.001	0.785	0.669
VB1 status						
VB1 sufficiency	1		1			
VB1 deficiency	4.816 (2.981-7.781)	<0.001	5.082 (2.211-11.677)	<0.001	0.762	0.531
VB7 status						
VB7 sufficiency	1		1			
VB7 deficiency	7.400 (3.655-14.982)	<0.001	7.313 (2.501-21.382)	<0.001	0.763	0.003
VC status						
VC sufficiency	1		1			
VC deficiency	5.907 (3.212-10.863)	<0.001	3.056 (1.089-8.578)	0.034	0.742	0.061

*Adjusted for age, temperature, respiratory rate, heart rate and neutrophil percentage.

MPP, *Mycoplasma pneumoniae* pneumonia, VA, vitamin A, VD, vitamin D, VB1, itamin B1, VB7, vitamin B7, VC, vitamin C.

## 4 Discussion

Vitamins are essential micronutrients vital for maintaining normal physiological functions in the human body, with significant roles in immune regulation. For instance, VA deficiency impairs immune function, increasing susceptibility to infections (19) Specifically, VA is critical for maintaining the integrity and function of respiratory mucosal epithelial cells, a key component of “mucosal immunity” (20). Given that *MP* initiates infection by adhering to respiratory epithelium, optimal VA status may theoretically reduce the risk of initial pathogen adhesion and colonization. Similarly, VD plays a pivotal role in modulating immune responses, and its deficiency has been linked to severe respiratory infections (21) Beyond its classical immunomodulatory functions, VD can induce the production of antimicrobial peptides (e.g., cathelicidin) (22)-, suggesting a potential direct antimicrobial role against various pathogens, which might extend to *MP*. More importantly, the immunopathology of MPP is characterized by excessive inflammatory responses (23). VD’s ability to modulate both innate and adaptive immunity, potentially mitigating this immunopathological damage, could influence disease severity and complications (10) This study found that children with MPP exhibited significantly lower levels of VA and VD compared to healthy controls, which aligns with previous research linking VA deficiency to pneumonia pathogenesis (24). Notably, the prevalence of VA deficiency in patients with MPP was 62.5% (25), considerably higher than in healthy children, underscoring its potential impact on respiratory health and immunity. Additionally, VD insufficiency has been associated with a higher occurrence of pneumonia in children, particularly in low-income countries, where affected children often experience more severe symptoms (26). Moreover, patients with MPP demonstrated significantly reduced levels of VB1, VB7, and VC, with higher deficiency rates than healthy controls, suggesting that these vitamins may also influence immune function and contribute to the development of MPP.

The mechanisms linking B vitamins (VB1, VB7) to infection are less specific. Their primary roles are as coenzymes in cellular energy metabolism (27). Severe deficiencies could lead to generalized metabolic dysregulation and impaired immunity, increasing overall susceptibility to infections (28). However, a direct, specific pathogenic link between isolated VB1 or VB7 deficiency and MPP remains speculative and is rarely explored in the literature. In contrast, VC is a potent antioxidant and immune supporter (29). VC, as a key antioxidant, may help alleviate this oxidative damage to lung tissue (30). It also supports various immune cell functions, potentially aiding the host response.

Correlation analyses revealed significant negative associations between vitamin levels and age, likely due to dietary imbalances during school-age development, leading to inadequate intake. Furthermore, our data showed that vitamin levels were negatively correlated with neutrophil and monocyte percentages but positively correlated with lymphocyte percentages, suggesting anti-inflammatory roles for these vitamins. Experimental evidence supports the notion that VA can suppress pro-inflammatory cytokine expression in macrophages and T-cells while increasing anti-inflammatory mediators like IL-10 and TGF-β (31). VD modulates immunity by inhibiting NF-κB signaling, reducing pro-inflammatory cytokines, and enhancing IL-10 production and regulatory T-cell (Treg) activity (32). Vitamin C is a crucial anti-inflammatory and antioxidant molecule that plays a vital role in various infectious diseases. Substantial evidence indicates an inverse correlation between vitamin C levels and inflammatory markers, such as C-reactive protein (33, 34). Supplementation with vitamin C significantly reduces the production of pro-inflammatory cytokines, underscoring its clinical value in both the prevention and treatment of respiratory infections, including influenza and COVID-19 (35–37). B vitamins also contribute to immunoregulation by modulating the functions of T cells and macrophages during inflammation (38). For instance, studies have shown that VB1 treatment decreases pro-inflammatory cytokine production, exhibits antioxidant properties, and protects immune cells from oxidative damage (39–41). Clinical trials further demonstrate that septic shock patients receiving VB1 experience increased ICU-free days (42, 43). However, evidence regarding VB1 supplementation’s benefits in septic shock, such as reduced mortality and renal protection, remains inconsistent. Similarly, VB7 influences the production of pro-inflammatory cytokines within the immune system (44). VB7 deficiency has been associated with elevated levels of cytokines including IL-1β, TNF-α, IFN-γ, and IL-17 (45–47). Despite these findings, few studies have explored the relationship between B vitamins, e.g., VB1, VB, and MPP. The precise mechanisms by which B vitamins modulate inflammatory responses and infectious diseases require further investigation. The observed associations for VB1 and VB7 in our study may reflect a state of generalized nutritional insufficiency impacting overall immune competence rather than a specific pathogenic role in MPP.

Vitamin plays a critical role in immune function, suggesting a potential link to the pathogenesis of MPP. Deficiencies in VA and VD have been linked to MPP in previous reports (25, 48, 49), which is consistent with the associations observed in this study. Children with inadequate levels of VA or VD had significantly higher odds of having MPP compared to those with sufficient levels. Of particular significance is the well-documented immunological synergy between vitamin A and D, which supports their coordinated role in immune regulation. Our analysis extends this understanding by demonstrating that their co-deficiency exhibits a substantially stronger association with MPP than individual deficiencies, suggesting a clinically relevant interaction that aligns with established biological mechanisms. In addition, deficiencies in VB1, VB7, and VC were also independently associated with the disease, with affected groups showing a higher prevalence of MPP. While the roles of VA and VD in respiratory infection pathogenesis are more clearly defined, the evidence for VB1, VB7, and VC in the specific context of MPP remains limited. Their contribution might be more supportive, through antioxidant effects (VC) or maintenance of general metabolic and immune health (B vitamins). These observed associations highlight the link between suboptimal vitamin status and increased susceptibility to respiratory infections, underscoring the importance of clinical monitoring and intervention in pediatric vitamin nutrition. The observed associations raise the possibility that targeted nutritional strategies might be beneficial, a premise that warrants further investigation. It is important to note that the observational nature of this case-control study means that the identified associations between vitamin levels and MPP do not establish causality. The possibility of reverse causality, wherein the infection itself may influence vitamin status, must be considered. Nevertheless, these findings robustly highlight a significant and independent relationship that merits further investigation in prospective studies.

This study provides the first comprehensive assessment of multiple vitamin profiles, particularly B vitamins, in children with MPP, revealing widespread deficiencies and their significant associations with infection susceptibility. However, several limitations should be considered when interpreting the results. First, the retrospective single-center design and the relatively small sample size may introduce selection bias and limit the generalizability of our findings. Therefore, future prospective, large-scale, multi-center studies are necessary for validation. Second, although our analysis adjusted for several confounders, important unmeasured factors-such as detailed dietary intake, lifestyle habits, anthropometric Z-scores, and socioeconomic status-could introduce residual confounding, which underscores the necessity of more comprehensive data collection in future research. Third, the spectrum of vitamins analyzed was constrained by the routine clinical testing panel available in our hospital. Notably, data on vitamin B12 levels were not available due to this limitation, despite its recognized importance in immune function. Additionally, the reference ranges for vitamin B7 were based on standards from the Mayo Clinic due to the absence of established population-specific reference values in China. This may have influenced the observed high prevalence of deficiency in both study groups and should be considered when interpreting these particular results. Future studies should aim to include a broader range of micronutrients, particularly vitamin B12, and establish population-appropriate reference ranges for accurate nutritional assessment. Finally, data on vaccination history (e.g., against influenza or pneumococcal disease) were not collected. Although no *MP*-specific vaccine exists, the potential influence of general immune status modulated by other vaccinations cannot be fully ruled out as a confounder.

## 5 Conclusion

In conclusion, this study demonstrates that children with MPP have significantly lower levels of VA, VD, VB1, VB7, and VC compared to healthy controls. These vitamins also show a significant negative correlation with neutrophil percentage in peripheral blood. Multivariate regression analysis identified VA, VD, VB1, VB7, and VC as factors independently associated with MPP.

## Data Availability

The original contributions presented in the study are included in the article/**Supplementary Material**. Further inquiries can be directed to the corresponding authors.

## Author disclaimer

All authors confirm that this manuscript is a transparent and honest account of the reported research. This work is related to a previously published study by the same author group, titled “Associations of single and multiple vitamin levels with pediatric oral mucosal diseases: a cross-sectional study with multi-model analysis “ (DOI: 10.3389/fnut.2025.1677164). Both studies are sub-analyses derived from the same overarching research project, “A Study Evaluating Vitamin Nutritional Status in Children of Henan Region Based on HPLC-MS/MS Detection Technology,” which is the reason for sharing the same ethical approval (No. 2022-K-L045). While both studies utilize the same core methodological approach for vitamin detection and stem from the same regional pediatric cohort project, they address fundamentally distinct clinical questions in non-overlapping patient groups. The published study investigated the association between vitamins and oral mucosal diseases. The current submission, “Serum Vitamins and Mycoplasma Pneumoniae Pneumonia in Children: A Case-Control Study,” investigates the relationship between serum vitamins and MPP, employing a specific case-control design and analyzing a completely independent dataset focused on respiratory infection.
