# Supplementary material for: Serum vitamins and Mycoplasma pneumoniae pneumonia in children: a case-control study
Source: Front Immunol. 2026 Feb 25;17:1676950. doi: 10.3389/fimmu.2026.1676950 (PMC12975596; doi:10.3389/fimmu.2026.1676950)
Supplement: Supplementary file 1 [file Table1.docx]

**Supplementary Table 1.** Association between the combined level and status of vitamin A and D and MPP.

| Variables | Univariate | |  |  | | Multivariate* | | |
| --- | --- | --- | --- | --- | --- | --- | --- | --- |
|  | OR (95% CI) | P |  | OR (95% CI) | P | | Nagelkerke R² | Hosmer-lemeshow test (P) |
| VA+VD (ng/ml) | 310.452  (102.757-937.943) | ＜0.001 |  | 380.724 (57.018-2542.182) | ＜0.001 | | 0.791 | 0.249 |
| VA and VD status |  |  |  |  |  | |  |  |
| VA and VD sufficiency | 1 |  |  | 1 |  | |  |  |
| VA and VD deficiency | 188.297  (67.139-528.101) | ＜0.001 |  | 101.494 (21.111-487.946) | ＜0.001 | | 0.804 | 0.790 |

Note: *Adjusted for age, temperature, respiratory rate, heart rate and neutrophil percentage.

Abbreviations: MPP, *Mycoplasma pneumoniae* pneumonia, VA, vitamin A, VD, vitamin D, VB1, vitamin B1, VB7, vitamin B7, VC, vitamin C.
